# HERE‐Bi: Feasibility and Acceptability of a Self‐Esteem Intervention for Young Bisexual People to Reduce Non‐Suicidal Self‐Injury

**DOI:** 10.1002/cpp.70117

**Published:** 2025-07-09

**Authors:** Jade Wilkinson, Peter J. Taylor, Brendan J. Dunlop

**Affiliations:** ^1^ Division of Psychology and Mental Health, Manchester, School of Psychological Sciences, Manchester Academic Health Sciences Centre University of Manchester Manchester UK

**Keywords:** affirmative, bisexuality, CBT, NSSI, self‐esteem, therapy, youth

## Abstract

**Background:**

Bisexual youth are at a disproportionate risk of non‐suicidal self‐injury (NSSI). Unique stressors, such as biphobia and bi‐erasure, and their impact on self‐esteem, identity and a sense of belonging may help explain this disproportionate risk. Affirmative therapies promote self‐acceptance and identity affirmation and may therefore be effective in boosting self‐esteem and reducing NSSI.

**Aims:**

This study aimed to assess the feasibility and acceptability of a cognitive behavioural therapy‐based, self‐esteem intervention for young bisexual people (aged 16–25) with experience of NSSI thoughts, urges and behaviour. Secondarily, this study examined indicators of change on measures of self‐esteem, NSSI, identity and belongingness.

**Methods:**

A case series design was used with eight participants who experienced NSSI urges, thoughts or behaviours in the previous 6 months. Participants received an affirmative, 8‐week, self‐esteem intervention and completed measures of self‐esteem, NSSI, belongingness and identity.

**Results:**

Six participants attended all eight therapy sessions, and two opted out before completion. There were no serious adverse events. The intervention was deemed to be acceptable and feasible, and evidence of improvements on measures of self‐esteem, NSSI, positive identity and thwarted belongingness was observed.

**Conclusions:**

The results suggest that this intervention is a safe, acceptable and feasible intervention, warranting further evaluation. Revisions and considerations for future trials of this affirmative therapy are proposed.

## SUMMARY

- A virtually delivered, one‐to‐one affirmative self‐esteem intervention is feasible, acceptable and safe for young bisexual people.
- Affirmative therapy that incorporates systemic considerations into formulations is helpful for individuals to differentiate between internalised biphobia and unhelpful thinking patterns.
- Interventions that are flexible and responsive to the needs of the participant aid engagement and perceived effectiveness.

## 1 Introduction

The definition of bisexuality has evolved over time (Mereish et al. 2017) and can mean different things to different people. A generally encompassing definition is ‘sexual and/or romantic attraction to more than one sex or gender’. Bisexual people are at a disproportionate risk of mental health difficulties compared to heterosexual and same‐sex‐attracted peers (Ross et al. 2014, 2018; Ruch and Bridge 2022; Russell and Fish 2016). They are also up to six times more likely to engage in non‐suicidal self‐injury (NSSI) compared to heterosexual, gay and lesbian peers (Dunlop et al. 2020; Irish et al. 2019; Taliaferro and Muehlenkamp 2017). Adolescence and early adulthood are developmentally crucial periods (Casey et al. 2010) when identity and sexuality are being explored (Arnett 2014; Moshman 2014) and NSSI incidence is high (Plener et al. 2015). NSSI has been defined as ‘the intentional destruction of one's own body tissue without suicidal intent’ (Klonsky et al. 2014, 565). There is a robust link between NSSI urges and behaviour (Hamza et al. 2015), and more intense urges have been shown to predict more frequent NSSI (Turner et al. 2019). There is debate about the value of distinguishing NSSI from suicidal behaviours. Although concerns have been raised about reliably establishing suicidal intent (Kapur et al. 2013), it has been argued that NSSI is distinct from suicidal behaviour in function, form and motive (Butler and Malone 2013), and there is evidence that participants see this behaviour as meaningfully distinct from suicide attempts (e.g., Taliaferro et al. 2019). Self‐esteem, defined as the evaluative or affective part of an individual's self‐concept (Leary and Baumeister 2000), has been linked with the risk of engaging in NSSI (Forrester et al. 2017; Taylor et al. 2018). In a sample of young bisexual people, self‐esteem was associated with NSSI, whereby higher levels of self‐esteem were linked to lower NSSI urge severity and lower risk of NSSI behaviour (Dunlop et al. 2022).

The mental health disparity experienced by bisexual people may be driven by stressors that are unique to sexual minority groups, including discrimination and hostility related to sexual identity (Brooks 1981), and the absence of social safety (i.e., reliable social connection, inclusion and protection; Diamond and Alley 2022). Unique stressors are likely to increase hypervigilance and lead to the internalisation of prejudice and negative societal attitudes (Meyer 1995, 2003). They may also experience intra‐minority stress from within the LGBTQ+ community bi‐negativity and bi‐erasure such as having their bisexual identity doubted or invalidated (Feinstein 2020; Israel and Mohr 2004; Parmenter et al. 2021), which can include being viewed as ‘disloyal’ to the gay or lesbian community for engaging in heterosexual relationships, leading to feelings of not belonging to any group (McLean 2008).

Therefore, it is intuitive that the internalisation of monosexist, biphobic narratives and thwarted belonging to and with others may negate the ability to form a positive bisexual identity (Bridge et al. 2022; Israel and Mohr 2004) and contribute to lower self‐esteem and NSSI (Assavedo and Anestis 2016; Chu et al. 2016; Gailliot and Baumeister 2007). Low self‐esteem and a lack of perceived belonging have been linked to NSSI in sexual minorities (Dunlop et al. 2022; Taylor et al. 2018) and may be an important mechanism leading to self‐injury in this population whilst forming a positive identity might be protective. Having a positive bisexual identity refers to one's sense of being a bisexual person and part of the wider community of bisexual people and recognising strengths and benefits of this (Riggle et al. 2014) and hence is a more specific construct than self‐esteem focused on a specific domain of self‐identity. A more positive bisexual identity may foster greater overall self‐esteem and perceived belongingness. Affirmative self‐esteem interventions may therefore hold the potential to reduce NSSI in young bisexual people, through enhancing self‐esteem, positive sexual identity and perceived belonging; however, societal bi‐invisibility also means that there is a lack of an affirmative community of support (Dodge et al. 2012; Hayfield et al. 2014; MacKay et al. 2017; Ross et al. 2010) and gaps in formal mental health support (Flanders et al. 2015).

A review by Calvo et al. (2022) highlighted a modest evidence base for psychological interventions targeting NSSI within adolescents; however, none are specifically tailored for bisexual young people. Without consideration of bisexual populations, these interventions may not address the proximal, distal and systemic factors that can contribute to the aetiology of NSSI (Marshall 2016; Smithee et al. 2019). Although we must ultimately aim to remove societal and systemic stressors (Dunlop and Lea 2023; Kitzinger 1997; Meyer 2020), support on an individual level can also be helpful as macro‐level changes take time (Bry et al. 2018; Gonzalez et al. 2021; Miller and Major 2000; Szymanski and Gonzalez 2020). There is evidence that therapy outcomes from psychological therapy are poorer for bisexual people (Rimes et al. 2018, 2019). Evidence of poorer outcomes for LGBTQ+ people has already prompted researchers and clinicians to adapt existing interventions to better meet the needs of this population (e.g., Craig, Eaton, et al. 2021; Hall et al. 2019; Lucassen et al. 2015), but we are aware of no adapted interventions for NSSI in bisexual people.

Affirmative practice is a way to enhance an existing model of treatment (Davies 1996; Pachankis 2018) by acknowledging and mitigating systematic barriers, promoting self‐acceptance, enhancing coping and strengths, exploring and validating positive expression of identity and considering the impact of macro‐level factors (e.g., monosexism, biphobia and bi‐erasure) on mental health (Langdridge 2007; Tozer and McClanahan 1999; O'Shaughnessy and Speir 2018; Van Den Bergh and Crisp 2004). Emerging evidence suggests that affirmative therapies may hold promise for improving mental health difficulties in sexual minority young people (Craig, Eaton, et al. 2021; Pachankis et al. 2015; Pachankis et al. 2022; Van Der Pol‐Harney and McAloon 2019). However, no affirmative interventions have targeted self‐esteem to reduce NSSI for bisexual young people.

This study therefore aimed to assess the feasibility and acceptability of a novel, 8‐week, self‐esteem intervention for young bisexual people: HERE‐Bi (Helping Raise Self‐Esteem in Bisexual Young People). This was a synchronous intervention delivered by a clinician online. Guidelines for developing an evidence base for complex interventions emphasise the importance of context and how existing interventions may need to be adapted to work effectively in a different context (Skivington et al. 2021). Investigating feasibility is an important phase in evaluating interventions, which can involve small‐scale studies initially (Skivington et al. 2021). Young people here refer to individuals aged 16–25, an age bracket commonly used in UK government legislation and previous studies with this population (Dunlop et al. 2022). This age range is a developmentally crucial time where identity is typically being explored (Arnett 2014). Feasibility refers to whether the study can practically be carried out, considering processes such as recruitment, intervention format and questionnaire completion. This was compared against progression criteria to determine if progression to a larger trial was warranted. Acceptability refers to whether the intervention feels appropriate and suitable for participants (UK Health Security Agency 2020). The secondary aim was to investigate changes in participants' ratings of self‐esteem, belongingness, positive identity, and NSSI thoughts, urges and behaviour changed following the intervention.

## 2 Methods

### 2.1 Design

A case series was conducted, whereby participants completed a series of questionnaires at baseline (1 week before the first session), midpoint (week four of the intervention) and follow‐up (1 week after the final session). Participants also completed a shorter, sessional measure at each intervention session. This intervention was initially designed to be a group intervention; however, due to recruitment challenges, it was amended to be an individual, one‐to‐one intervention (see Appendix A). All individuals who had signed up for the group intervention were informed and consented to receive the intervention on a one‐to‐one basis. The research design and intervention were reviewed and discussed with bisexual people who offered feedback, which is important given that only around half of the interventions created for LGBTQ+ populations engage the LGBTQ+ community in intervention design (Fowler et al. 2023; see Supplementary File S1 for further information). This research received ethical approval from University of Manchester Research Ethics Committee 2 (2023‐15264‐32242).

### 2.2 Participants

To be eligible to take part, individuals needed to (1) be aged 16–25 years old; (2) identify as bisexual or be attracted to more than one gender; (3) have experienced NSSI thoughts, urges or behaviours in the previous 6 months; (4) have a device and Internet access to be able to access the Zoom video call service; and (5) be in the United Kingdom. Individuals were not eligible if they were currently undergoing other psychological intervention, were unable to understand spoken English (due to time and budget limitations) or had current plans or intentions to end their life or engage in medically serious self‐injury, which may result in accidental death or hospital admission. Current NSSI behaviour did not exclude individuals from participating, and a comprehensive risk protocol with input from individuals with lived experience was developed to manage risk arising within the study.

Multiple recruitment methods were used, including placing adverts around a university campus and on social media and emails to charities, LGBTQ+ youth groups and university counselling services who shared details of the study with their contacts. This also included seeking out LGBTQ+ youth groups and charities specific to people of colour (POC) to try and gain a representative sample.

### 2.3 Intervention Development

The intervention was developed based on Melanie Fennell's self‐esteem model, which is grounded in cognitive behavioural therapy (CBT; Fennell 1997, 2006). CBT grounded in affirmative practice can be effective in helping minority groups acknowledge which stressors are internal and which are external, linked to oppressive societal factors (Wandrekar and Nigudkar 2019). Previous interventions based on Fennell's self‐esteem model have demonstrated significant improvements in self‐esteem (Kolubinski et al. 2018), and CBT‐based interventions have also demonstrated efficacy in improving various aspects of mental health and well‐being in sexual minority groups (Craig, Eaton, et al. 2021; Hart et al. 2021; Pachankis et al. 2022).

Affirmative aspects of this intervention followed various guidelines for affirmative therapy, including the 10 components of affirmative CBT (Craig et al. 2013). This also included understanding and recognising the effects of stigma and the unique experiences of bisexual individuals, validating individuals' unique strengths and supporting individuals to connect with a supportive community (American Psychological Association 2012; Carvalho et al. 2022; Feinstein et al. 2019), as well as linking specific bisexual experiences (e.g., biphobia) to self‐esteem development and maintenance, if relevant. As the presenting difficulty cannot be assumed to be linked to bisexuality (American Psychological Association 2012), this was not always the focus of the intervention, but minority stress and acknowledgement of systemic factors were interweaved throughout.

Participants were given a task to complete between weekly sessions as a meaningful part of the change strategy (Cronin et al. 2015). Each session started with a reflection on the previous session and the between‐session task and ended with the option of a mindfulness exercise (Carvalho et al. 2022). Individuals with lived experience were consulted to provide feedback on the intervention and changes were made to incorporate this (see Table 1 for intervention outline).

**TABLE 1:** Intervention outline.

Session	Description	Between‐session task
Session 1	What is low self‐esteem and where does it come from? Introduction of self‐esteem model (Fennell 1997). Exploration of minority stress and circles of influence (Dunlop 2022). Completion of longitudinal formulation	Reflect on session and make any relevant additions to longitudinal formulation
Session 2	What keeps low self‐esteem going? Considering impact of biphobia and the impact on self‐esteem and anxious thoughts/feelings. Discussion and completion of the anxious pathway	Worksheet to identify anxious predictions and precautions
Session 3	Checking out anxious predictions using cognitive (Catch it, Check it, Change it; Dunlop 2022) and behavioural (experiments) strategies	To try out behavioural experiments using a recording worksheet
Session 4	What keeps low self‐esteem going? Discussion and completion of the depressed pathway. Exploration of biphobia and impact on self‐esteem, self‐criticism and depressed thoughts/feelings	Spotting and combatting self‐criticism worksheet
Session 5	Identifying strengths and positive qualities and including them in the formulation. Exploring identity and values. Consideration of bisexual/LGBTQ+ role models	Add to a ‘My Strengths’ log. Daily activities diary
Session 6	Considering the impact of rules for living and finding new rules. Considering how to put new rules into practice	Try out experiments to put new rules into practice and record on worksheet
Session 7	Revisiting the bottom line and considering a new bottom line. Exploring experiments with the new bottom line	Try out experiments with the new bottom line and record on worksheet
Session 8	Planning for the future. Summarising the intervention and learning points and creating a ‘Keeping Well Plan’	

### 2.4 Measures

#### 2.4.1 Feasibility and Acceptability

Feasibility data related to recruitment, therapy adherence (number of sessions attended) and study retention (outcome measures completed). The progression criteria were predetermined and refined by the research team and experts‐by‐experience (Avery et al. 2017; see Table 2) using a red, amber, green (RAG) system to indicate suitability for progression onto larger trials (‘red’ = issues that cannot be remedied, ‘amber’ = some remediable issues prior to progression onto a larger trial, ‘green’ = feasibility for progression onto a larger trial). This approach to progression criteria has been used in previous studies with similar targets (Nielsen et al. 2023; Potter et al. 2021).

**TABLE 2:** Progression criteria for feasibility.

Indicator	Green	Amber	Red
Recruitment: number of participants recruited within 6 months	10–12	6–9	< 5
Therapy adherence: proportion of participants attending at least five out of eight sessions	> 70%	50%–70%	< 50%
Study retention: proportion of participants completing outcome measures at each data collection point (baseline, mid‐point and follow‐up)	> 70%	50%–70%	< 50%

A feedback questionnaire was created to gain acceptability feedback based on the Theoretical Framework of Acceptability (TFA) constructs (Sekhon et al. 2017, 2022; see Appendix B). Questions relate to seven domains: affective attitude, burden, perceived effectiveness, intervention coherence, self‐efficacy, opportunity costs and general acceptability. The TFA constructs also included ‘ethicality’, that is, the extent to which the intervention was a good fit with an individual's value system. However, a question relating to this construct was not included as participants have reported struggling to answer ethicality‐related questions previously (Sekhon et al. 2022). The questionnaire contained 10 Likert‐scale questions, whereby higher scores indicated greater acceptability, and 10 open questions for qualitative feedback (see Supplementary File 3). Acceptability was also assessed by reviewing attendance at sessions, completion of questionnaires and the number of serious adverse events (SAEs).

#### 2.4.2 Demographics

A demographic questionnaire was administered at baseline to record participant age, gender, sexuality, ethnicity, employment status and marital status. Owing to the number of ways to define bisexuality (Swan 2018), multiple options were provided, in addition to a free‐text box, to allow participants to self‐identify.

#### 2.4.3 Self‐Injury Urges

Self‐injury urges were measured using the Alexian Brothers Urge to Self‐Injure Scale (ABUSI; Washburn et al. 2010), a five‐item questionnaire measuring urges to self‐injure in the previous week. Scores can range from 0 to 30, whereby higher scores denote more intense urges. The ABUSI demonstrates good validity and reliability (*a* = 0.92–0.96; Dunlop et al. 2022; Washburn et al. 2010), including in a previous study of NSSI with young bisexual people (Dunlop et al. 2022). The ABUSI was administered at baseline, mid‐point, and follow‐up.

#### 2.4.4 NSSI

NSSI was measured using five questions from the NSSI component of the Self‐injurious Thoughts and Behaviours Inventory Short Form (SITBI‐SF; Nock et al. 2007). The SITBI‐SF demonstrated reliability and validity with adolescents and young adults (Nock et al. 2007) and has been used in a previous study of NSSI with young bisexual people (Dunlop et al. 2022). The SITBI‐SF examined the occurrence, nature and frequency of NSSI behaviour. To avoid extreme guesses, questions about the frequency of NSSI were asked on a Likert scale (Brown et al. 2022). This measure was administered at baseline, mid‐point and follow‐up.

#### 2.4.5 Self‐Esteem

The Internal Protective subscale of the Suicide Resilience Inventory‐25 (SRI‐25; Osman et al. 2004) was used to assess self‐esteem. The subscale contains nine items; scores range from 9 to 54, with higher scores indicating higher self‐esteem. This SRI‐25 subscale has been used to measure self‐esteem in studies on self‐harm and sexual orientation, with support for its factor structure and reliability (*α* = 0.94; Dunlop et al. 2022; Taylor et al. 2018). This subscale was administered at baseline, mid‐point and follow‐up.

#### 2.4.6 Thwarted Belongingness

The Thwarted Belonging subscale of the Interpersonal Needs Questionnaire (INQ; Van Orden et al. 2012) was used to measure thwarted belongingness. It is an eight‐item subscale with total scores ranging from 7 to 56, whereby higher scores indicate greater thwarted belonging. This subscale has demonstrated good reliability and convergent validity with related constructs (*a* = 0.86; Dunlop et al. 2022; Hill et al. 2015; Van Orden et al. 2012). This subscale was administered at baseline, mid‐point and follow‐up.

#### 2.4.7 Positive Identity

The Authenticity subscale of the Lesbian, Gay and Bisexual Positive Identity Measure (LGB‐PIM; Riggle et al. 2014) was used to examine authenticity as a measure of positive identity. The five‐item subscale total score ranges from 5 to 35, whereby higher scores indicate higher levels of positive identity. It has demonstrated good internal consistency and validity within LGBTQ+ youth populations (*α* = 0.88–0.92; Cooke and Melchert 2019; Harrison 2021; Riggle et al. 2017). This subscale was administered at baseline, mid‐point and follow‐up.

#### 2.4.8 Sessional Questionnaire

In order to minimise participant burden, a sessional questionnaire was created to reduce the number of questionnaires participants were required to complete at each session (Santangelo et al. 2013; see Supplementary File 2). This four‐item measure was created to monitor NSSI urges and behaviour during the intervention, in addition to monitoring contact with support services. Participants completed this questionnaire before each intervention session.

### 2.5 Procedure

Individuals self‐referred to the study and were screened for eligibility via video call before completing an online consent form. The first therapy session was arranged with a researcher at a mutually convenient time. One week prior to the first therapy session, participants received a link and QR code via email to complete the baseline measures on Qualtrics. Participants then attended one 60‐min session per week with the researcher, a trainee clinical psychologist, via video call for 8weeks. The researcher delivering the intervention received weekly supervision with a clinical psychologist experienced in LGBTQ+ mental health intervention. Participants were sent sessional measures on the morning of each session to complete beforehand, the mid‐point measures at Week 4 and the follow‐up measures 1 week after completion of the final intervention session. At the end of the intervention, participants were given a £5 shopping voucher per attended session to reimburse them for their time and effort.

### 2.6 Analysis

As this was an acceptability and feasibility trial and was therefore not powered to detect statistically significant changes, no formal statistical analyses were conducted on these outcomes. Missing data were excluded listwise. Mean change in scores (and associated 95% confidence intervals) were calculated as well as the paired samples Cohen's *d* (calculated as the average change divided by the standard deviation of the change). The Reliable Change Index (RCI) was also calculated for participants on measures of NSSI urges, self‐esteem, thwarted belongingness and positive identity, denoting the number of participants demonstrating reliable change on these measures (Ferrer and Pardo 2014; Guhn et al. 2014); RCIs exceeding 1.96 were deemed to be reliable. Qualitative data were analysed using a brief thematic analysis to identify themes between participant opinions of the intervention (Braun and Clarke 2006).

## 3 Results

### 3.1 Participant Characteristics

**FIGURE 1:**
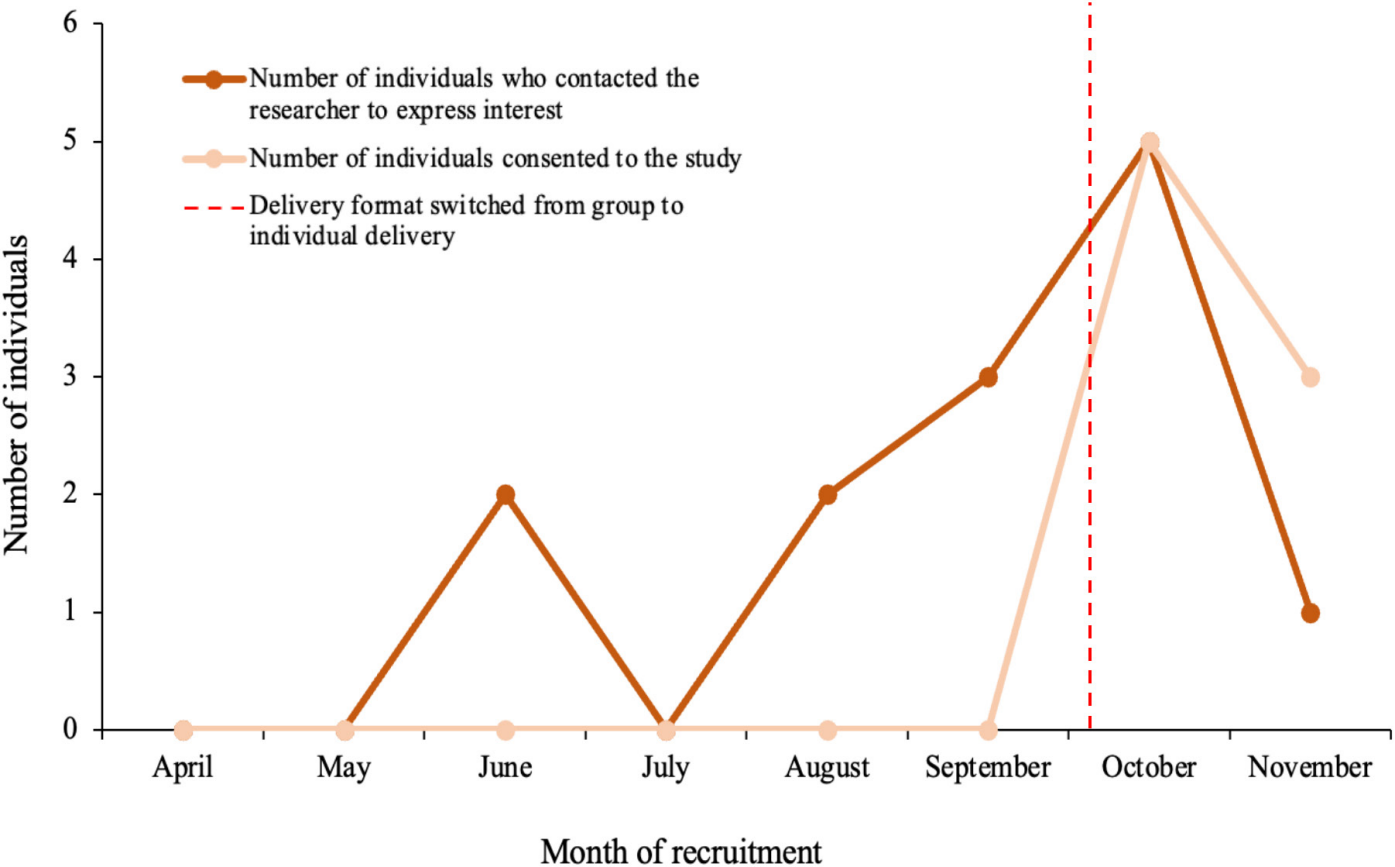
Rates of recruitment from April to November 2023.

**FIGURE 2:**
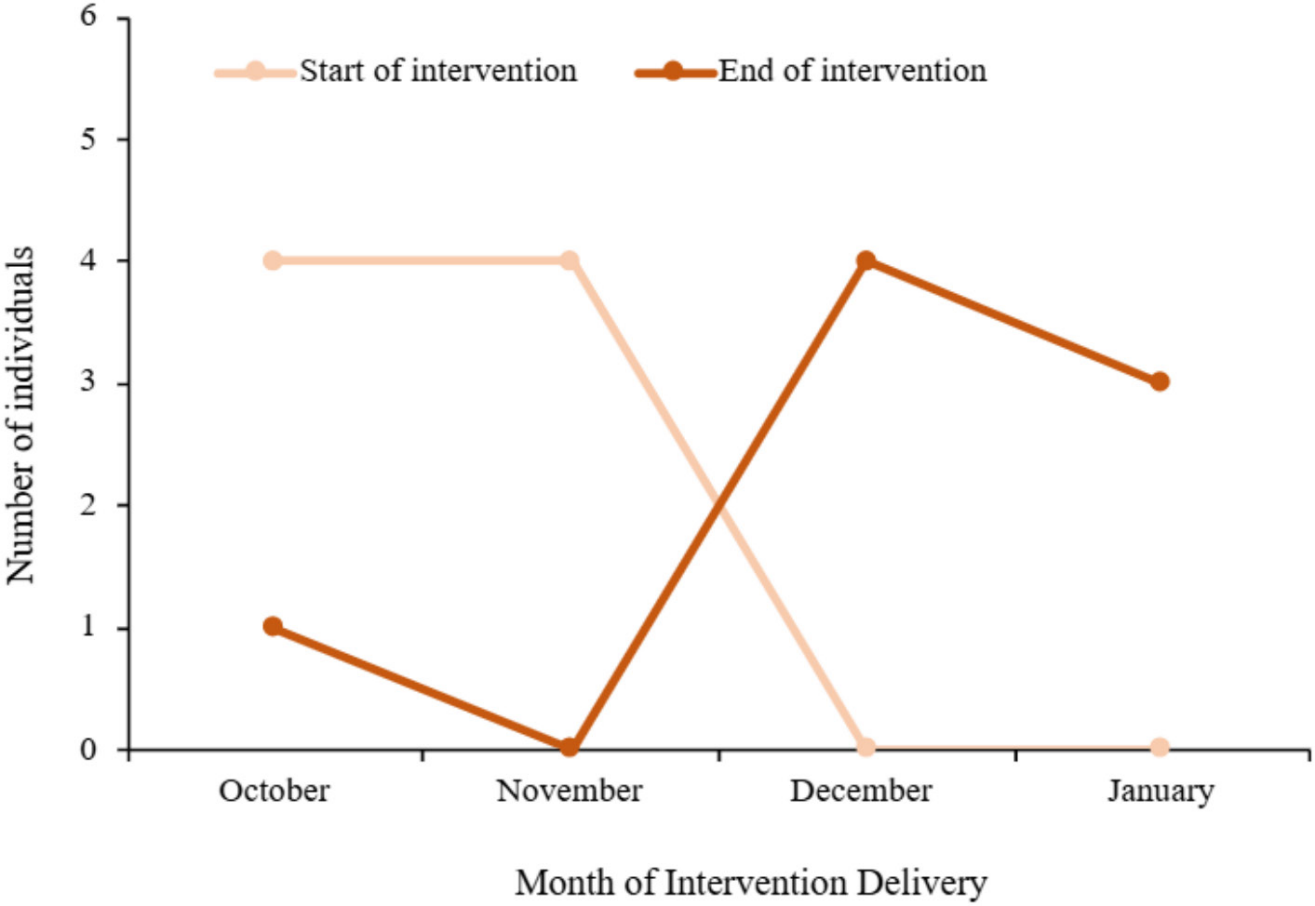
Number of participants starting and ending the intervention from October 2023 to January 2024.

Participants were recruited from April 2023 until November 2023 (see Figure 1). Eight participants took part in the study from October 2023 to January 2024 (see Figure 2). Ages ranged from 17 to 25, and the majority of participants were transgender (75%) and White British in ethnicity (75%). The most common forms of NSSI, as reported in the SITBI‐SF, were cutting or carving skin and hitting oneself on purpose. At baseline, most participants (62.5%) reported 0–5 episodes of hurting oneself or wanting to die in the past month and 11–30 episodes (50%) in the past year. Further demographic details are provided in Table 3.

**TABLE 3:** Sample characteristics at baseline.

Variable	Mean (SD)/frequency (%)
Age	21.75 (2.92)
Gender	
Male	3 (37.5)
Female	1 (12.5)
Non‐binary/third gender	3 (37.5)
Gender fluid	1 (12.5)
Gender the same as sex assigned at birth?	
Yes	2 (25)
No	6 (75)
Sexuality	
Bisexual	8 (100)
Ethnicity	
Asian/Asian British: Pakistani	1 (12.5)
White British	6 (75)
Other mixed/multiple ethnic background: South African	1 (12.5)
Highest level of education completed	
College	4 (50)
Undergraduate degree	2 (25)
Master's degree	2 (25)
Employment status	
Employed full‐time	3 (37.5)
Employed part‐time	2 (25)
Student	3 (37.5)
Marital status	
Married	2 (25)
Single	5 (62.5)
Partnered	1 (12.5)
Forms of NSSI	
Hit yourself on purpose	7 (87.5)
Cut or carved skin	7 (87.5)
Scraped your skin to the point of drawing blood	5 (62.5)
Bit yourself	5 (62.5)
Burned your skin	3 (37.5)
Punching walls	1 (12.5)
Inserted objects into your skin or nails	1 (12.5)
Episodes of NSSI in the past month	
0–5	5 (62.5%)
6–10	3 (37.5%)
Episodes of NSSI in the past year	
0–5	1 (12.5%)
6–10	2 (25%)
11–30	4 (50%)
31–50	1 (12.5%)

*Note:* Participants selecting ‘Other mixed/multiple ethnic background’ were provided a textbox to self‐describe their ethnicity.

### 3.2 Feasibility

Therapy adherence and study retention were indicated to be feasible for progression onto a larger trial (see Table 2 for progression criteria). Six participants completed all eight sessions of the intervention. One participant opted out after completing session two because they had reached the top of a waiting list for CBT for social anxiety with another organisation, and another participant opted out after session four with no reason provided. Study retention was 75%, with six participants providing data at all three data points. Recruitment was highlighted as ‘amber’, suggesting some remediable issues prior to progression onto a larger trial. As reflected in Figure 1, recruitment rates were low when the study was advertised as a group intervention from April to October 2023. However, when the intervention format changed to one‐to‐one therapy, eight participants were recruited within 6 weeks.

### 3.3 Acceptability

All participants completing the feedback questionnaire (*n* = 7) rated HERE‐Bi as being ‘acceptable’ or ‘completely acceptable’. Feedback indicated that the intervention was perceived to improve self‐esteem and reduce NSSI thoughts, urges and behaviour and that it was clear how the intervention did so (see Appendix B for summary). Completion of the questionnaires suggests that the questionnaire frequency and length were acceptable to participants. Similarly, no participants suggested that questionnaires were burdensome or problematic. There were no SAEs during the study.

Qualitative feedback indicated that most participants (*n* = 5) felt that 60‐min sessions were sufficient, though they would have preferred more sessions to overcome initial nerves and allow for more in‐depth exploration of content and consolidation. Participants found the recruitment process ‘simple’ and ‘straightforward’ and cited therapeutic models, worksheets, mindfulness, being online and therapist interpersonal factors as contributing to the effectiveness of sessions. When asked about their experience of sessions, most participants described making conceptual links as helpful for gaining insight and understanding of themselves and their patterns of behaviours (*n* = 5).

I never thought I had any internalised biphobia before, but doing this made me realise I do.

You don't need to have low self‐esteem to be a good person. I always thought that I had to feel really down about myself all the time to make sure I was keeping myself and my ego in check, but you can just be proud of yourself and like yourself as well as being kind and a good person.

Boosting self‐love and acceptance, ending sessions with mindfulness and a focus on identity and mapping out wider systemic influences were also highlighted as important parts of their experience.

I feel like a bisexual man doesn't really have a space. I feel like people need me to either be gay or straight.

Several barriers to engagement were reported, including finding time and appropriate space to attend and technological challenges when using a smartphone to attend, and one participant reported struggling with the rigidity of the CBT approach at times. Participants suggested that future delivery of HERE‐Bi could be improved by being in‐person, consolidation of worksheets so there are fewer to avoid confusion and interweaving strengths‐based activities throughout all sessions. Three participants felt it would have been helpful to know whether the facilitator was part of the LGBTQ+ community to know how much context to provide around their experiences and potentially establish a better rapport. However, others (*n* = 3) felt that this did not impact on effectiveness because they were ‘well educated on LGBTQ+ terms and experiences’ and provided a space that felt ‘understanding’ and ‘compassionate’.

### 3.4 Changes in Outcomes

When considering average changes, all outcomes improved from baseline to follow‐up with the exception of positive identity (see Table 4). Most participants demonstrated a reduction in the frequency of NSSI thoughts (*n* = 5), the intensity of NSSI urges (*n* = 6), time spent thinking about NSSI (*n* = 6), difficulty resisting NSSI (*n* = 5) and urges in the previous week (*n* = 6). One participant showed an increase in NSSI urge intensity at the final data point, which was uncharacteristic of the trends in the rest of their data, and one participant who did not complete the intervention reported a slight increase in the time spent thinking about NSSI (from less than 20 min to 21–45 min).

**TABLE 4:** Average scores and changes on outcome measures.

Measure	Baseline M (SD)	Mid‐point M (SD)	Follow‐up M (SD)	Baseline to follow‐up Cohen's *d* (95% CI)	Baseline to mid‐point Cohen's *d* (95% CI)	Mid‐point to follow‐up Cohen's *d* (95% CI)
ABUSI	11.88 (5.08)	8.5 (7.84)	4 (3.16)	1.34 (−2.72–5.4)	0.37 (−5.88–6.62)	0.75 (−3.41–4.91)
SRI‐25	28.5 (7.37)	35 (4.75)	40.5 (7.21)	−1.38 (−7.36–4.6)	−0.79 (−6.52–4.94)	−1.18 (−4.41–2.05)
INQ	30.63 (6.97)	30.5 (7.89)	23.88 (7.49)	0.85 (−4.67–6.37)	0.02 (−3.91–3.95)	1.01 (−3.56–5.58)
LGB‐PIM	27.25 (3.33)	27.75 (5.73)	29.75 (5.60)	−0.38 (−4.96–4.2)	−0.08 (−4.71–4.55)	−1.22 (−2.36 to −0.08)

**FIGURE 3:**
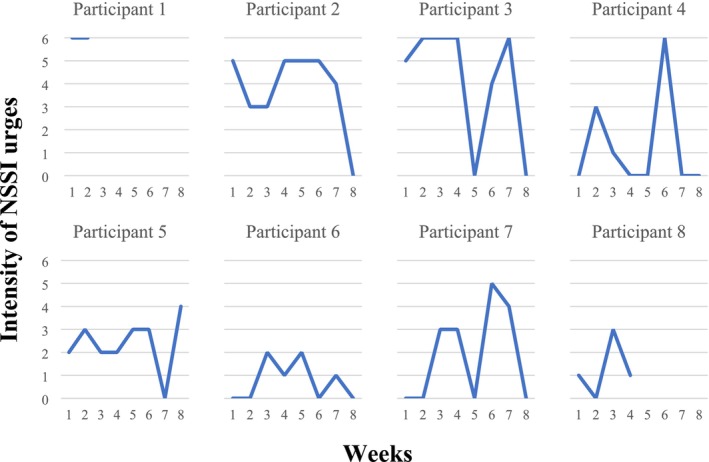
Intensity of participant's self‐injury urges throughout the intervention. *Note:* 0 = *not applicable (no urges)*, 1 = *very mild urge*, 2 = *mild urge*, 3 = *moderate urge*, 4 = *strong urge, easily controlled*, 5 = *strong urge, difficult to control*, 6 = *strong urge, would have self‐injured if able to*.

Self‐injurious behaviour and urges were assessed throughout the study using the sessional questionnaire. As demonstrated in Figure 3, self‐injury urges fluctuated over the course of the intervention; however, they typically demonstrated maintenance or a reduction from the first to the final session. Two participants contacted support services during the intervention.

### 3.5 Reliable Change

Six participants demonstrated a reliable improvement in self‐esteem scores from baseline to follow‐up. Five participants showed a reliable improvement in ratings of NSSI urges, and four participants showed a reliable improvement in thwarted belongingness (one reliable deterioration) and positive identity scores.

## 4 Discussion

This study aimed to explore the acceptability and feasibility of a novel, self‐esteem intervention for young bisexual people who self‐injure and, secondarily, explore changes in outcome measures. The findings indicate that this eight‐session, CBT‐based intervention was feasible and acceptable to participants. The progression targets were largely met, and there were no SAEs, suggesting that the intervention was safe and that further evaluation of the approach is warranted.

The feasibility of the intervention was reflected in study retention rates and therapy adherence. The intervention had a 25% attrition rate. Of participants completing the intervention, 100% completed all study measures, and one participant who opted out completed the follow‐up questionnaire. The majority of participants reported high levels of satisfaction with the intervention. Participant feedback emphasised the importance of incorporating wider, systemic considerations into formulations in order to highlight problematic societal structures and narratives and differentiate between internalised biphobia and unhelpful thinking patterns. Intervention feedback also highlighted the need to be person‐centred and flexible with the session content. For instance, there were themes of self‐compassion running through some session discussions that have been found to buffer the effects of discrimination and improve self‐esteem (Helminen et al. 2023; Thomason and Moghaddam 2021). Self‐acceptance also emerged as a theme through some sessions, and both of these components were highlighted as an important part of what made the sessions effective. This is consistent with other research highlighting the importance of self‐acceptance within LGBTQ+ groups (Camp et al. 2020, 2022) and its relevance to effective therapy for self‐injury (Haw et al. 2023). For others, strictly following the CBT‐based model offered a helpful structure, and mindfulness was reported to be a valuable adjunct to therapy to provide containment and re‐orientation prior to ending the session, which is congruent with mindfulness literature (Carvalho et al. 2022).

An amber rating on the recruitment target indicated that further consideration of recruitment is warranted prior to progression onto further trials (Avery et al. 2017). It is possible that recruitment took time to build momentum as the number of individuals expressing an interest increased over time, which should be factored into the time budgets of future trials. It was also likely impacted by the intervention initially being advertised as a group, which was hypothesised to be the most beneficial delivery format given the links between thwarted belongingness and NSSI in this population (Dunlop et al. 2022). However, the low rate of interest in the intervention when advertised as a group may indicate that this delivery format was less popular amongst young bisexual people. It is possible that a group format felt less safe or more exposing to this population given the stigma that exists around both bisexuality and NSSI (Chen et al. 2022; Meheli and Banerjee 2022; Pitt et al. 2024), but further research is needed to confirm if this was the case. Considerations for setting up and offering group‐based interventions for LGBTQ+ people are discussed in more detail elsewhere (Camp et al. 2023; Cohen et al. 2021). Future trials exploring this intervention will therefore likely ascertain a more accurate recruitment rate based on the one‐to‐one intervention. There are also some additional recruitment strategies that may be piloted in future trials to enhance reach, based on previous studies of similar interventions and populations (Craig, Eaton, et al. 2021; Dunlop et al. 2022). Specifically, an outreach aspect, such as visiting youth groups, and a digital strategy, such as videos on social media, may legitimise the study and familiarise potential participants with the study and researchers. Relatedly, how this group is advertised may be important to consider for future trials. Although it is important to tailor support for specific marginalised groups to hold their experiences in mind, it is also worth considering whether advertisements exclude potentially eligible individuals. For instance, individuals who conceal their identity or do not adopt the ‘bisexual’ label due to different philosophical stances regarding labelling identity. This is particularly pertinent given that bisexual people are most likely to conceal identity (Feinstein et al. 2020) and young people currently, Gen Z, are moving towards rejecting labels or adopting more general labels such as ‘queer’ (Hammack et al. 2022), highlighting the need to consider within‐group differences just as we consider between‐group differences (Lee and Hobbs 2022).

Improvements in ratings of self‐esteem and thwarted belongingness were observed, which is promising given self‐esteem was the main, direct target of the intervention. These changes cannot be taken as evidence of efficacy on their own as the current study was not designed for this purpose. A statistically powered exploration of these effects, with a control group and randomisation, would be a helpful next step to better understand this relationship, whether these changes can be attributed to the intervention.

The results of this study are encouraging; however, there are some important considerations and modifications to be carried forward that may improve the intervention. Preferences for face‐to‐face versus online delivery are likely to vary dependent on factors such as geographical location, familial support and ‘outness’, thus offering an option in this regard based on individual preference is likely to increase accessibility (Craig, Iacono, et al. 2021; McDermott et al. 2016, 2024). To accommodate a person‐centred approach, elective modules could be created to guide therapists in being formulation‐driven. For instance, having acceptance‐ and compassion‐ focused approaches for young people whose formulations indicate that this would be beneficial. Although the target group for this intervention was bisexual young people, 75% of the sample were transgender and/or non‐binary. It is unclear why such a larger proportion of trans people were recruited, but it may reflect the level of need in this group given NSSI is also prevalent amongst the trans community (Liu et al. 2019) and the lack of access to care. Future research could explore how different aspects of identity, including gender, interact with the therapy experience through an intersectional lens.

### 4.1 Limitations

There are several limitations that should be taken with these findings. The lack of a control or comparator condition means that we cannot determine whether change in outcomes was attributable to the intervention or other, uncontrolled variables. Further, the effect sizes should be interpreted with caution as they are likely to be inflated by the small sample and design (Parker et al. 2020), and thresholds are arbitrary (Thompson 2007). Alternative small N designs, such as multiple baseline designs, whilst still limited by small numbers and a subsequent lack of precision, may have allowed for a stronger initial test of clinical promise. However, in line with the aims of this study, we were able to examine the feasibility of the intervention's processes and procedures in preparation for a larger, controlled trial (Eldridge et al. 2016), which is now needed to confirm whether these effects are the results of the intervention and are replicable on a larger scale. Additional recruitment considerations centring around POC are warranted for future trials. POC participants are under‐represented in bisexual literature (Ghabrial and Ross 2018), and ethnicity can impact therapeutic outcomes (Kivlighan et al. 2019). Therefore, more targeted outreach to gain representation in future, larger scale trials is crucial for wider intersectionality considerations. Although the present study did not include a measure of fidelity to the model, this would be beneficial in future trials examining efficacy. However, fidelity to the protocol should be carefully balanced with using professional judgement to offer a person‐centred intervention.

This study tested several aspects of feasibility, but other feasibility uncertainties that would be relevant for a subsequent randomised controlled trial (RCT), such as the ability to collect data in a control group, were not assessed, and so further piloting and feasibility testing is warranted. Self‐report measures may have advantages when assessing stigmatised behaviours like NSSI (Fox et al. 2020) but may also introduce bias and could be supplemented with other forms of assessment (e.g., review of medical records) in future research. The follow‐up period was relatively short, and as such, it is unclear how outcomes may have changed in the longer term.

Despite these challenges, this study indicates that this intervention holds promise for improving self‐esteem and reducing NSSI in young bisexual people. Based on the encouraging findings from this initial study, further investigation of efficacy is now warranted. Further trials should explore whether feasibility is maintained in a RCT design, then explore the intervention's efficacy with a larger, sufficiently powered sample (Eldridge et al. 2016). This should include a longer follow‐up period to explore whether any significant changes are sustained.

### 4.2 Therapeutic Considerations

The key findings from this study indicate that further evaluation of the intervention is warranted. However, in the absence of a robust evidence‐base for specific affirmative interventions, there are also some learning points that practitioners may wish to draw from this study. The formulation of difficulties should include systemic factors and wider, external considerations to acknowledge the role of minority stressors (as in Dunlop 2022), such as the role of a biphobic workplace culture. It is crucial to validate these experiences, and the individual's response to these (perhaps in the context of minority stress theory [Brooks 1981; Meyer 1995, 2003], where relevant and appropriate) prior to any change‐related or resilience‐building work. Any change mechanisms for presenting difficulties should incorporate previously highlighted systemic differences, with a focus on building personal strengths and resilience to mitigate systemic factors that are outside an individual's control. These suggestions are in line with recommendations for culturally sensitive therapeutic practice and developing safe therapeutic spaces for LGBTQ+ people (Beattie and Allán 2024). Acceptance and compassion‐based activities can also be helpful to support individuals in managing difficulties in the face of things they cannot control. In the example used above of the influence of a biphobic workplace culture, this could be accepting that an individual cannot control what others think or do, but they can control how they personally respond.

## 5 Conclusion

This study has shown promise for the feasibility, acceptability and safety of an 8‐week, one‐to‐one, CBT‐based intervention in improving self‐esteem and reducing NSSI risk in young bisexual people. These results further extend support for the use of affirmative CBT and support the rationale for the implementation of a larger scale study to test the efficacy of this intervention. Future research should focus on continuing to develop and evaluate the evidence base for affirmative interventions with a focus on the visibility of bisexual young people, building on the current promise of this evidence base in removing accessibility barriers and improving current mental health trajectories for young bisexual people.

## Conflicts of Interest

The authors declare no conflicts of interest.

## Supporting information


**Data S1** Supporting Information.

## Data Availability

The data that support the findings of this study are available from the corresponding author upon reasonable request.
