# Supplementary material for: HERE‐Bi: Feasibility and Acceptability of a Self‐Esteem Intervention for Young Bisexual People to Reduce Non‐Suicidal Self‐Injury
Source: Clin Psychol Psychother. 2025 Jul 9;32(4):e70117. doi: 10.1002/cpp.70117 (PMC12239551; doi:10.1002/cpp.70117)
Supplement: Supplementary file 1 — Data S1 Supporting Information. [file CPP-32-e70117-s001.docx]

**Appendix A** – Discussion of intervention changes

Initially, the study was intended to be delivered in a group at the University of Manchester with a view to foster social connectedness and improve thwarted belongingness, however there was not enough interest in the study after three months of recruitment efforts. The intervention was therefore then advertised as an online intervention to remove the geographical barrier, however there was still insufficient interest. The contingency plan of a 1-to-1 intervention was therefore actioned after a further three months, and a sufficient number of participants was recruited within six weeks.

**Appendix B** – Summary of average acceptability feedback scores across all participants

| **Question**  Likert scale ranges | **M (SD)** |
| --- | --- |
| Did you like or dislike HERE-Bi?  0 = strongly dislike  5 = strongly like | 4.86 (0.35) |
| How much effort did it take to engage in the HERE-Bi intervention?  0 = no effort at all  5 = huge effort | 2.57 (1.05) |
| HERE-Bi has improved my self-esteem  0 = Strongly disagree  5 = Strongly agree | 4.43 (0.73) |
| HERE-Bi has reduced my non-suicidal self-injury urges  0 = Strongly disagree  5 = Strongly agree | 3.86 (0.64) |
| HERE-Bi has reduced my non-suicidal self-injury behaviour  0 = Strongly disagree  5 = Strongly agree | 4 (0.53) |
| It is clear to me how HERE-Bi helps improve self-esteem  0 = Strongly disagree  5 = Strongly agree | 4.57 (0.49) |
| It is clear to me how HERE-Bi helps reduce non-suicidal self-injury urges/behaviour  0 = Strongly disagree  5 = Strongly agree | 4 (0.86) |
| Engaging in HERE-Bi interfered with my other priorities  0 = Strongly disagree  5 = Strongly agree | 1.86 (0.41) |
| How confident did you feel about engaging in HERE-Bi?  0 = Very unconfident  5 = Very confident | 4 (0.93) |
| How acceptable was HERE-Bi to you?  0 = Completely unacceptable  5 = Completely acceptable | 4.86 (0.35) |

**Supplementary File 1** – Intervention Consultation Process

At the point of idea conception, a member of the Community Liaison Group at the University of Manchester and LGBTQ+ charities were consulted on the ideas (specifically, Hidayaj LGBT+, BiPhoria and 42^nd^ Street). They gave feedback on the intervention idea, recruitment considerations, readability of recruitment posters, completion format of questionnaire measures and delivery format, which was incorporated into the study.

Once the intervention had been finalised, prior to the start of the intervention, a member of the Community Liaison Group and BiPhoria were again consulted for their thoughts and input on the intervention, including session content, questionnaires and format. Feedback at this point related to between-session work, rapport-building and setting the scene for the intervention with participants.

**Supplementary File 2** – HERE-Bi Sessional Questionnaire

Have you had urges to self-injure in the past week?  Yes  No

On average, how strong have the urges been?

 **Slight**, that is, a very mild urge.

 **Mild** urge.

 **Moderate** urge.

 **Strong** urge, but **easily** controlled.

 **Strong** urge, but **difficult** to control.

 **Strong** urge **and would have self-injured if able to**.

Have you engaged in self-injury in the past week?  Yes  No

If so, how many times? ___________

Have you contacted any support services (e.g. Samaritans, YoungMinds) in the past week?

 Yes  No

If so, how many times? ___________

**Supplementary File 3** – HERE-Bi Feedback Questionnaire

HERE-Bi Feedback Questionnaire

1. Did you like or dislike HERE-Bi?

| Strongly dislike | Dislike | No opinion | Like | Strongly like |
| --- | --- | --- | --- | --- |
| 1 | **2** | **3** | **4** | **5** |

2. How much effort did it take to engage in the HERE-Bi intervention?

| No effort at all | A little effort | No opinion | A lot of effort | Huge effort |
| --- | --- | --- | --- | --- |
| 1 | **2** | **3** | **4** | **5** |

3. HERE-Bi has improved my self-esteem

| Strongly disagree | Disagree | No opinion | Agree | Strongly agree |
| --- | --- | --- | --- | --- |
| 1 | **2** | **3** | **4** | **5** |

4. HERE-Bi has reduced my non-suicidal self-injury urges

| Strongly disagree | Disagree | No opinion | Agree | Strongly agree |  |
| --- | --- | --- | --- | --- | --- |
| 1 | **2** | **3** | **4** | **5** | **N/A** |

5. HERE-Bi has reduced my non-suicidal self-injury behaviour

| Strongly disagree | Disagree | No opinion | Agree | Strongly agree |  |
| --- | --- | --- | --- | --- | --- |
| 1 | **2** | **3** | **4** | **5** | **N/A** |

6. It is clear to me how HERE-Bi helps improve self-esteem

| Strongly disagree | Disagree | No opinion | Agree | Strongly agree |
| --- | --- | --- | --- | --- |
| 1 | **2** | **3** | **4** | **5** |

7. It is clear to me how HERE-Bi helps reduce non-suicidal self-injury urges/behaviour

| Strongly disagree | Disagree | No opinion | Agree | Strongly agree |
| --- | --- | --- | --- | --- |
| 1 | **2** | **3** | **4** | **5** |

8. How confident did you feel about engaging in HERE-Bi?

| Very unconfident | Unconfident | No opinion | Confident | Very confident |
| --- | --- | --- | --- | --- |
| 1 | **2** | **3** | **4** | **5** |

9. Engaging in HERE-Bi interfered with my other priorities

| Strongly disagree | Disagree | No opinion | Agree | Strongly agree |
| --- | --- | --- | --- | --- |
| 1 | **2** | **3** | **4** | **5** |

10. How acceptable was HERE-Bi to you?

| Completely unacceptable | Unacceptable | No opinion | Acceptable | Completely acceptable |
| --- | --- | --- | --- | --- |
| 1 | **2** | **3** | **4** | **5** |

11. Please tell us in as much detail as possible about your experience of attending the sessions? (e.g., how helpful was it overall? What did you make of it?)

12. Do you think that 60-minutes is an appropriate amount of time for the HERE-Bi sessions?

13. There were eight sessions of the intervention. How did that number of sessions feel (i.e. would you have preferred less/more)?

14. Was there anything that you feel made the sessions effective?

15. Do you have any recommendations for improving the HERE-Bi intervention?

16. Were there any barriers or challenges to you attending the HERE-Bi intervention?

17. What are your thoughts on the facilitator and their identity?

18. How did you find the recruitment process?

19. If there is any other feedback you would like to provide which hasn’t yet been asked about, please provide it here:

**Supplementary File 4** – Additional quotes from feedback questionnaire

Experiences of attending the sessions:

“It was certainly helpful to see how my thoughts feed into each other and explore how early experiences have influenced who I am now. Looking at model was also an incredibly useful and insightful way to see how my thoughts look when written down, as it was clear to see where patterns were.”

“Overall the sessions really helped me to understand myself better and why I respond to things the way I do. The insight has been very helpful.”

“The sessions have been extremely helpful as they helped me to understand why I was feeling the way I was. One main reason in why it was so helpful if we went through the theory behind how I can change my behaviours and having that additional knowledge helped me in seeing the overall process.”

“It was a huge help to look at myself in a way that frames my identity as the focus and map out how I view myself and others based on how I am influenced and how I interact with the world in different ways. It has helped me see how I follow destructive pathways and bring out both positive and negative aspects of myself in any situation. This has hugely helped me understand my actions and let me learn to sit with myself as a whole not as fragments that can be used or taken out of context. The sessions were well structured and the mindfulness was a wonderful way to end the session.”

“I found repeating certain exercises and processes extremely helpful. Getting into a routine of self-love and acceptance is something I’ve not thought about doing or been able to do before. I’m really glad someone was there to help initiate that.”

“I found the sessions very helpful with sorting thoughts that I had already been having into a more logical visual layout with the help of the models and visual aids. It helped me spot patterns and themes in regards to my struggles and what I value which formed a more cohesive understanding of the undercurrents of my mental health. At times, the rigidity of the models were difficult to fit my exact thoughts and experiences into, however it was rewarding to notice the emerging themes. Moreover, sometimes just saying things out loud which I'd never told anyone and having a good cry was cathartic enough without the CBT framework. I felt a sense of progression through the sessions and I was interested to experience therapy both as a first timer and as a psychology student.”

Session duration:

“Absolutely. Any longer and the focus would be lost/ could have been overwhelming.”

“I think 90-120 minutes may be better, frequently found that there was not enough time to cover all desired topics while also keeping to the treatment schedule. Having it be a non-fixed end time would also help with”

“Yes, there were some sessions where I could have said more but definitely well-timed for most of them as they are emotionally, mentally and physically draining.”

Number of sessions:

“I feel more sessions would've been better as he first few sessions I was still on edge”

“8 sessions was a very good length i feel like more or less would be needed depending on the individual. Personally, i would have liked an additional session as i got off track often and we ran out of time to finish the last session work. And a consolidation excersise would have been nice, but im truly being pedantic about that.”

“More sessions, felt rushed trying to get through all of the session materials”

“Although it was challenging to let go, 8 sessions made a large impact. I would have done more sessions if I could. Already knowing the length of the course impacted my ability to share certain things; I went less into detail in certain areas because I didn't want to leave loose ends. It was a good introduction to the therapeutic experience though.”

Effective elements:

“The structure and the diagrams of the model was very effective to see how the therapy was building up and working through was very good. Also Jade is an excellent therapist.”

“Going through models and talking through my ideas and thoughts.”

“How understanding Jade was and how working with her helped me change my perspective on certain situations.”

Recommended improvements:

“Maybe giving more space to build up or revisit the positives throughout the sessions so that when the positives session comes around the patient is used to looking for the good qualities and the excersise is able to be done jn more depths?”

“If it were in person it may run slightly smoother.”

“Perhaps too many worksheets. It got a little confusing”

Barriers:

“Family didn’t understand and since I live at home it was difficult to find quiet spaces to attend the sessions.”

“Sometimes my phone or laptop was not working so it made it hard to see the documents and sometimes we had to change the day and times.”

“I work odd hours so schedulling was sometimes interesting.”

“Apart from being over Christmas, so timings made it difficult but Jade was very flexible and forgiving. Being on zoom in both my student house and at home is not free from the worry of being overheard, so in-person may be a better delivery style.”

“As mentioned, trying to squeeze my experiences into some of the models was hard, and sometimes I couldn't think of answers to questions/boxes that had been worded in particular ways, e.g. my bottom lines. Sometimes these were just hard to say out loud”

Facilitator identity:

“Excellent delivery of the therapy. Whilst I'm not sure and didn't ask much of their identity, this does not get in the way of the effectiveness. All that was needed was a person who is able to listen and nudge patients in the right direction.”

“I would have liked to know if the facilitator was queer before we began, however they were extremely patient and compassionate and wise. I am so grateful.”

“The facilitator was very understanding and kind. I was able to be relaxed with them.

It would be helpful to know if the facilitator is part of the LGBT+ community as it would give me understanding of how much they know about the community as a whole and how much context to give to situations.”

“Jade was very professional and supportive. She'd never make assumptions and helped tie things that I've said into the themes I'd already laid out eg control, being embarrassed of my identity, the importance of community etc. I didn't know if Jade was queer or not, so I wasn't sure how relatable what I was saying is. It would have built better rapport/deeper connection perhaps if I'd known (but I wouldn't want to be intrusive)”

**Supplementary File 5** – Clinician Details:

At the time of the study, Dr Jade Wilkinson was a 3^rd^ year Trainee Clinical Psychologist. She had worked in the NHS since 2018, with experience in primary mental health services, inpatient, forensic, child and adolescent and older adult services.

Dr Brendan J Dunlop and Dr Peter J Taylor have published numerous papers on bisexual mental health, including a focus on bisexual women’s experiences of psychological therapy, bisexual young people’s experiences of NSSI and a meta-analysis on NSSI risk for bisexual people. Dr Brendan J Dunlop has also published The Queer Mental Health Workbook (2022) and the intersectional/social/systems framework with Dr James Lea and he works clinically with bisexual clients.

In preparation for the study, the lead author engaged in various continuing professional development activities related to bisexual mental health. This included consultation and engagement with young bisexual people, and attending mental health lectures relating to LGBTQ+ issues and minority stress theory. The two senior authors have conducted extensive prior research on the mental health of bisexual populations, and research supervision focussed on these specific experiences and learnings for the lead author. Finally, the lead author received weekly clinical supervision from an experienced clinical psychologist trained in LGBTQ+ mental health. These mechanisms all allowed for honest reflection in research and clinical supervision on any biases, blind spots and feelings towards the intervention and research project, to mitigate any implicit bias.
